# Validation of the Provincial Transfer Authorization Centre database: a comprehensive database containing records of all inter-facility patient transfers in the province of Ontario

**DOI:** 10.1186/1472-6963-6-129

**Published:** 2006-10-06

**Authors:** Victoria A Robinson, Russell D MacDonald, Doug Manuel, Vivek Goel

**Affiliations:** 1 Institute of Medical Science, University of Toronto, Toronto, Canada; 2 ORNGE – Transport Medicine, Toronto, Canada; 3 Division of Emergency Medicine, Department of Medicine, University of Toronto, Toronto, Canada; 4 Institute for Clinical Evaluative Sciences, Toronto, Canada; 5 Department of Public Health Sciences, University of Toronto, Toronto, Canada; 6 Department of Health Policy, Management and Evaluation, University of Toronto, Toronto, Canada

## Abstract

**Background:**

The Provincial Transfer Authorization Centre (PTAC) was established as a part of the emergency response in Ontario, Canada to the Severe Acute Respiratory Syndrome (SARS) outbreak in 2003. Prior to 2003, data relating to inter-facility patient transfers were not collected in a systematic manner. Then, in an emergency setting, a comprehensive database with a complex data collection process was established. For the first time in Ontario, population-based data for patient movement between healthcare facilities for a population of twelve million are available. The PTAC database stores all patient transfer data in a large database. There are few population-based patient transfer databases and the PTAC database is believed to be the largest example to house this novel dataset. A patient transfer database has also never been validated. This paper presents the validation of the PTAC database.

**Methods:**

A random sample of 100 patient inter-facility transfer records was compared to the corresponding institutional patient records from the sending healthcare facilities. Measures of agreement, including sensitivity, were calculated for the 12 common data variables.

**Results:**

Of the 100 randomly selected patient transfer records, 95 (95%) of the corresponding institutional patient records were located. Data variables in the categories patient demographics, facility identification and timing of transfer and reason and urgency of transfer had strong agreement levels. The 10 most commonly used data variables had accuracy rates that ranged from 85.3% to 100% and error rates ranging from 0 to 12.6%. These same variables had sensitivity values ranging from 0.87 to 1.0.

**Conclusion:**

The very high level of agreement between institutional patient records and the PTAC data for fields compared in this study supports the validity of the PTAC database. For the first time, a population-based patient transfer database has been established. Although it was created during an emergency situation and data collection is dependent on front-line medical workers, the PTAC data has achieved a high level of validity, perhaps even higher than many purpose built databases created during non-emergency settings.

## Background

Patient transfers between health care facilities are an essential component of any health care system. Inter-facility patient transfers occur for routine to urgent reasons. For example, transfers are arranged for imaging studies, clinic appointments, or specialized treatment. In the province of Ontario, Canada with its population of twelve million there are approximately half a million inter-facility patient transfers annually.

Despite the great volume of patient movement due to inter-facility transfers, these movements were not tracked, monitored or documented in a systematic manner. The Severe Acute Respiratory Syndrome (SARS) outbreak in Ontario in 2003 necessitated the surveillance of patient movement activities. Although there are many reasons for the spread of SARS between hospitals in the Toronto area, inter-facility patient transfers have been shown to be, in part, responsible for spread during the first and second wave of the outbreak [1,2].

The Provincial Transfer Authorization Centre (PTAC) was operational within days of the Ontario Ministry of Health and Long-Term Care suspending all patient inter-facility transfers. Established during a public health emergency and fully operational on April 1, 2003, PTAC continues to authorize, monitor and document all patient transfers in Ontario [3]. The PTAC receives between 1,100 and 1,500 transfer authorization requests each day. The PTAC was established as part of the emergency response to the SARS outbreak to monitor patient movement in the Ontario health care system. As part of its due diligence to protect the health of the public, in February 2004 the Ontario government announced that PTAC would become a permanent part of the provincial health care system.

Before 2003, data relating to inter-facility patient transfers were not collected in a systematic manner. For the first time in Ontario, population-based data for patient movement between health care facilities are available. The PTAC database stores patient information for every inter-facility patient transfer and transfer request regardless of approval status. The PTAC is believed to be the largest population-based patient transfer database in existence.

The data contained in the PTAC database offer a chance to examine inter-facility patient transfers comprehensively at the provincial level for the first time. There are very limited population-based studies of inter-facility patient transfers. Understanding the dynamics of these transfers will provide the foundation to better quantify the impact patient transfers have on the health care system and patient care, including their heavy cost burden.

Administrative data is being used more extensively in health services research. The validation of these databases is an important step in determining the quality of their data before they are used for research purposes. There are various approaches to data validation and these are highlighted in the medical literature [4-13]. This paper employs a gold standard, the institutional patient record, in the validation process. As such, the validation of the PTAC database will include accuracy levels for variables being validated and sensitivity measures.

## Methods

### Inter-facility patient transfer authorization process

There are several steps in the Ontario inter-facility patient transfer authorization process. First, the sending facility is required to complete a patient transfer authorization form. Health care facilities are encouraged to dedicate individuals to receive training from the PTAC on proper completion and submission of the transfer forms to ensure consistency. Front-line medical workers, usually nurses, complete the transfer authorization forms. The forms can be completed in paper format and faxed or online via a secure website. Once the transfer authorization form has been submitted to the PTAC, a medical decision algorithm is used to process the request. Research found that the medical decision algorithm used by PTAC had a very high sensitivity and high specificity during the Toronto SARS outbreaks [14]. When a transfer request is approved, it is assigned a transfer authorization number. The sending facility then books the transfer with the appropriate transfer service. If a transfer request does not pass the decision algorithm, it is reviewed by the PTAC on-call physician. The physician may override the algorithm, particularly in cases of medical necessity. The information contained in the completed PTAC transfer form is then entered into the PTAC database.

### The PTAC database

Shortly after PTAC implementation, a dedicated database was created in a matter of days to archive all patient transfer requests. The PTAC database now captures all data fields contained in the patient authorization form and the unique transfer authorization number, if the transfer is approved.

From a research perspective, the PTAC database contains the data necessary to perform epidemiologic studies, based on patient demographic and clinical information that could not be accomplished previously. The PTAC captures all requests for inter-facility patient transfers, including private transfers that are not routinely captured by Emergency Medical Systems, the National Ambulatory Care Reporting System, or hospital discharge data.

### Sampling process for validation

The validation process included selecting a random sample of 100 patient transfer records from the PTAC database and comparing these records to the institutional patient record for the transferred patient from the sending healthcare facility. The sample was selected based on the sending facility location.

A stratified random sample of 10 health care facilities was chosen in the Greater Toronto Area (GTA) and the process was completed to ensure sampling from high and low volume sending facilities, academic and non-academic centres and nursing home or long-term care facilities. At each of the 10 selected facilities, 10 randomly selected patient transfers occurring between June 1, 2004 and August 31, 2004 were selected for comparison during this validation process. A total of 100 patient transfers were examined.

The GTA was selected due to its availability of a large variety of health care facilities, its high volume of inter-facility patient transfers, and the fact that the PTAC was implemented in response to a GTA-based health care emergency.

Healthcare facilities were stratified into 8 categories: large, medium and small transfer volume teaching hospital; large, medium and small transfer volume community hospital; or medium and small transfer volume nursing home/long term care facility. Large transfer volume facilities referred to facilities that transferred more than 1,000 patients to another facility in a single year. Small transfer volume facilities referred to facilities that transferred less than 400 patients to another facility in a year and medium volume senders between 400 and 999 patient transfers (Table 1).

Once the 10 GTA facilities were identified according to the transfer volume selection criteria, 10 random patient transfers were selected within the sample timeframe using a random number generator. The PTAC record for each patient transfer was then compared to the institutional patient record of the sending facility for that patient. Twelve variables were collected and then compared for agreement.

### Sample size calculation

A sample of 100 was selected because it would allow detectable differences in error rates between the PTAC forms and the institutional patient records as small as 10% with 95% confidence. The validation sample size was calculated based on an estimate of 400,000 inter-facility patient transfers per year. Sample size calculations were performed using EpiInfo (version 3.3.2; CDC, Atlanta).

### Validation process

The institutional patient record has been used extensively as a gold standard to validate external databases. For the validation of a patient transfer database, the institutional patient record is the only data source that contains patient information from the same time period. The institutional patient record, therefore, was chosen as the gold standard for the validation.

There is also extensive literature on the quality of administrative data and the validation processes to ascertain their quality. Most of the validation processes contain level of accuracy measures, as well as, sensitivity analyses. In keeping with these methodologies, this validation study will present these measures.

A single researcher (VR) conducted the entire chart review eliminating errors stemming from multiple abstractors. The PTAC record was compared to the institutional patient record for each patient from the sending facility. Taking the patient record from the sending facility as the gold standard, the PTAC report fields were compared to the institutional patient record and all corresponding data fields were assessed for their accuracy. The sending facility institutional patient record, instead of the patient record from the accepting facility, was selected as the gold standard because it is at that point during the transfer that the data contained in both sources should be identical.

Data variables to be validated included patient last name, first name, age, sex, sending facility, receiving facility, medical supervision during transfer, transfer service employed, whether the transfer was emergent, urgent or non-urgent, transfer status and the primary reason for the transfer. The two free text variables, primary reason for transfer and transfer service, were deemed to be an exact match with the PTAC form if the information was consistent with the institutional patient record. If a detailed account of the transfer was not present in the institutional patient record, then an exact match could not be determined.

The variables were divided into four categories: facility identification and timing of transfer; patient demographics; transfer supervision and service; and reason and urgency of transfer. Table 2 summarizes the data fields on the PTAC form that are common with the institutional patient record.

Since the institutional patient record was taken as the gold standard, level of accuracy and sensitivity measures are the most appropriate validation processes. Accuracy levels were calculated using accuracy rates. Accuracy rate refers to the number of times a variable from the PTAC database matches the variable in the institutional patient record exactly divided by the sample size. Error rate refers to the number of times a variable from the PTAC database does not match the institutional patient record exactly divided by the sample size. The accuracy and error rate numerators do not include missing data. Missing data was examined separately.

The sensitivity was calculated to evaluate the accuracy of each variable when compared to the institutional patient record. Sensitivity accounts for agreement and disagreement levels when compared to the gold standard together in one measure.

## Results

Of the 100 institutional patient records sought in this validation process, 95 (95%) were located. The remaining 5 could not be located by the institutions' medical records departments. For all 5 missing medical records, there was a record of the patient having been at the institution on the date of the transfer through electronic medical record systems, but the physical paper medical record that contained all the data necessary for the validation was missing and declared lost by the institution.

Three out of the four data variable categories had strong accuracy rates and sensitivity measures. Facility identification and timing, demographics and reason and urgency of transfer categories all had variables with high rates of accuracy. One variable category was not consistently used – the category of transfer supervision and service. The variables medical supervision and transfer service were missing 80% of the time from either the PTAC form, institutional patient record or both. Medical supervision and transfer service were rarely recorded in the institutional patient record.

Accuracy rates for all 12 variables ranged from 17.9% to 100%. Disregarding the category of transfer supervision and service, the remaining 10 variables had very strong accuracy rates that ranged from 85.3% to 100%. Corresponding error rates ranged from 0 to 8.4%. The highest error rate was for the last name variable (Table 3).

Sensitivity was calculated for all of the variables. The results are contained in Table 4. All variables had strong sensitivity values ranging from 0.92 to 1.0.

## Discussion

Before any data source can be used with confidence, it should be validated against a gold standard. In this case, the gold standard was the institutional patient record from the sending facility and it was compared to a completed PTAC form for an inter-facility patient transfer. 95% of the institutional patient records were located for validation. Consistently used data variables (10) had very high agreement rates (85.3% to 100%). These variables also had strong sensitivity values (0.92 to 1.0).

The data variable categories of facility identification and timing, demographics and reason and urgency of transfer all had variables that had consistently strong accuracy rates and sensitivity values.

The highest overall error rate (8.4%) was for the last name variable and this was almost always due to spelling or typographical errors.

When 12 matching data variables from the PTAC forms and the institutional patient records were compared, one variable category was not consistently used. Medical supervision and transfer service information was hardly ever recorded in the institutional patient record and it was often absent from the PTAC record as arrangements for the actual transfer are usually made subsequent to approval for the patient transfer from PTAC. As a result, these data variables had low agreement rates and high rates of missing data. These variables, however, performed well during the sensitivity analysis demonstrating that when the variables were in fact used that the data in the PTAC record matched the data in the institutional patient record.

Accuracy rates for the other 10 variables were strong. The sensitivity values for the variables were also strong ranging from 0.87 to 1.0. The strong positive results from all of these measures of association demonstrate that there is a high level of validity for the PTAC data.

Despite a reliance on data capture from diverse sources, namely front-line medical workers, and its establishment during a healthcare emergency, the PTAC database still achieves high levels of accuracy. As a result, the PTAC database can be used as a legitimate data source for population-based research. This paper also presents a unique example of using institutional patient records as a gold standard to validate an external database.

## Conclusion

The very high level of accuracy between institutional patient records and the PTAC data for fields compared in this study indicate data contained in the PTAC database are valid. Future research plans for the PTAC database include modelling the relationship of the spread of disease to patient movement among hospitals using Monte Carlo simulation, the linking of patient transfer data to hospital outcome databases and other data sources and modelling patient movement throughout the healthcare system.

## Abbreviations

SARS: Severe Acute Respiratory Syndrome

PTAC: Provincial Transfer Authorization Centre

GTA: Greater Toronto Area

## Competing interests

The author(s) declare that they have no competing interests.

## Authors' contributions

VR conceived of the study, participated in the design of the study, performed all of the patient hospital chart abstraction, validated the data, drafted the manuscript and incorporated edits. RDM conceived of the study, acquired the PTAC data for validation and participated in the design of the study. DM conceived of the study and participated in the design of the study. VG conceived of the study and participated in the design of the study. All authors read and approved the final manuscript.

## Pre-publication history

The pre-publication history for this paper can be accessed here:



## Figures and Tables

**Table 1:** Stratification of healthcare facilities

Number of transfers (during the sample period)	Representation of total number of transfers during entire study period	Ideal relative number of facilities to be sampled (out of 10)	Total number of facilities in sampling frame	Number of teaching facilities to be sampled	Number of community facilities to be sampled	Number of nursing homes/long term care facilities to be sampled
Large volume senders (more than 1,000 patient transfers during the sample period)	50%	5	20	2	2	0*
Medium volume senders (between 400 and 999 patient transfers during the sample period)	21%	2	11	1	1	1
Small volume senders (between 1 and 399 transfers during the sample period)	29%	3	152	1	1	1

*No available facilities

**Table 2:** List of PTAC data fields to be validated

**Field**	**Description**
***Facility identification and timing of transfer***	
Sending facility	Health care facility sending the patient
Receiving facility	Health care facility receiving the patient
Date of transfer	Date of the patient transfer
	
***Patient demographics***	
Last name	Last name of transfer patient
First name	First name of transfer patient
Sex	Sex of transfer patient
Age	Age of the patient
	
***Transfer supervision and service***	
Medical supervision during the transfer	Whether medical supervision is required during the transfer (Y/N)
Transfer service	Service employed to complete the patient transfer (free text)
	
***Reason and urgency of transfer***	
Emergent/urgent/non-urgent	Was the patient transfer emergent, urgent or non-urgent?
Transfer status	Approval status for transfer (Y/N)
Primary reason	Primary reason for transfer (free text)

**Table 3:** Accuracy and error results

**Variable**	**Accuracy rate (%), (95% CI)**	**Error rate (%)**	**Missing data**		
			
			**Missing from PTAC (%)**	**Missing from institutional patient record (%)**	**Missing from both (%)**
***Facility identification and timing***					
Sending facility	100.0, (100.0–100.0)	0.0	0	0	0
Receiving facility	91.6 (87.9–95.2)	3.2	0	5.3	0
Date of transfer	85.3 (83.0–87.5)	1.1	0	13.7	0
					
***Demographics***					
Last name	91.6 (86.0–97.2)	8.4	0	0	0
First name	95.8 (91.7–100.0)	4.2	0	0	0
Sex	94.0 (90.1–97.3)	3.2	3.2	0	0
Age	91.0 (79.6–93.1)	1.1	5.3	0	0
					
***Transfer supervision and service***					
Medical supervision	17.9 (13.2–22.6)	1.1	9.5	22.1	49.5
Transfer service	20.0 (15.5–24.5)	1.1	6.3	21.1	51.6
					
***Reason and urgency of transfer***					
Emergent/urgent/non-urgent	90.5 (90.5–90.5)	0.0	0	9.5	0
Transfer status	90.5 (90.5–90.5)	0.0	0	9.5	0
Primary reason	95.8 (92.9–98.7)	2.1	0	2.1	0

**Table 4:** Sensitivity measures

**Variable**	**Sensitivity (95% CI)**
***Facility identification and timing***	
Sending facility	1.00 (1.00 – 1.00)
Receiving facility	0.97 (0.94 – 1.00)
Date of transfer	0.99 (0.97 – 1.00)
	
***Demographics***	
Last name	0.92 (0.87 – 0.98)
First name	0.96 (0.92 – 1.00)
Sex	0.97 (0.94 – 1.00)
Age	0.99 (0.97 – 1.00)
	
***Transfer Supervision and Service***	
Medical supervision	0.99 (0.97 – 1.00)
Transfer service	0.95 (0.91 – 0.99)
	
***Reason and Urgency of Transfer***	
Emergent/urgent/non-urgent	1.00 (1.00 – 1.00)
Transfer status	1.00 (1.00 – 1.00)
Primary reason	0.98 (0.95 – 1.00)
